# Heat shock factor 1-mediated transcription activation of Omi/HtrA2 induces myocardial mitochondrial apoptosis in the aging heart

**DOI:** 10.18632/aging.102361

**Published:** 2019-10-18

**Authors:** Dan Liu, Linguo Wu, Ye Wu, Xin Wei, Wen Wang, Suli Zhang, Ming Yi, Jing Li, Huirong Liu, Xinliang Ma

**Affiliations:** 1 Department of Physiology and Pathophysiology, Yan Jing Medical College, Capital Medical University, Beijing 101300, China; 2 Department of Pathology, Beijing LuHe Hospital of Capital Medical University, Beijing 101100, China; 3 Department of Physiology and Pathophysiology, School of Basic Medical Sciences, Capital Medical University, Beijing 100069, China; 4 Beijing Key Laboratory of Metabolic Disturbance Related Cardiovascular Disease, Beijing 100069, China; 5 Department of Cardiology, XuanWu Hospital Capital Medical University, Beijing 100053, China; 6 Department of Emergency Medicine, Thomas Jefferson University, Philadelphia, PA 19107, USA

**Keywords:** age-related pathology, mitochondria, transcriptional regulation, cardiovascular, Omi/HtrA2

## Abstract

Background: Increased cardiac apoptosis is a hallmark of the elderly, which in turn increases the risk for developing cardiac disease. The overexpression of Omi/HtrA2 mRNA and protein contributes to apoptosis in the aged heart. Heat shock factor 1 (HSF1) is a transcription factor that binds to the promoter of Omi/HtrA2 in the aging myocardium. However, whether HSF1 participates in cardiomyocyte apoptosis via transcriptional regulation of Omi/HtrA2 remains unclear. The present study was designed to investigate whether HSF1 plays a role in Omi/HtrA2 transcriptional regulation and myocardial apoptosis.

Methods and Results: Assessment of the hearts of mice of different ages was performed, which indicated a decrease in cardiac function reserve and an increase in mitochondrial apoptosis. Omi/HtrA2 overexpression in the elderly was negatively correlated with left ventricular function after exercise overload and positively correlated with myocardial Caspase-9 apoptosis. Chromatin immunoprecipitation (ChIP) of aging hearts and plasmid transfection/RNA interference of H9C2 cells revealed that enhancement of HSF1 expression promotes Omi/HtrA2 expression by inducing the promoter activity of Omi/HtrA2 while also increasing mitochondrial apoptosis by upregulating Omi/HtrA2 expression.

Conclusions: HSF1 acts as a transcriptional factor that induces Omi/HtrA2 expression and Caspase-9 apoptosis in aged cardiomyocytes, while also decreasing cardiac function reserve.

## INTRODUCTION

Aging is a major cardiovascular risk factor [1]. Cardiomyocyte apoptosis increases during aging, which in turn has been shown to be related to decreased cardiac reserve [2] and increased sensitivity to ischemia reperfusion injury [3], stress overload, and other cardiovascular diseases [4]. Studies have shown that mitochondria play an important role in regulating apoptosis during aging [5]. However, the molecular mechanism of mitochondrial-mediated apoptosis in the aging heart remains unclear.

Omi/HtrA2 is a pro-apoptotic protein located in the mitochondria, which is released into the cytoplasm after an apoptotic stimulus [6], initiating apoptosis [7]. Our preliminary study found that Omi/HtrA2 is released from the mitochondria and into the cytoplasm during myocardial ischemia-reperfusion (I/R) injury in rats, which in turn increases ischemia-reperfusion injury [8].

Our preliminary study found increased Omi/HtrA2 mRNA and protein expression in the myocardium of aging rats, aggravating I/R injury by inducing the apoptosis of myocardial cell [9]. Cardiac-specific overexpression of mitochondrial Omi/HtrA2 promotes cardiomyocyte apoptosis and heart dysfunction [10], activates Caspases, and disrupts mitochondrial homeostasis in the aged heart [11]. However, the regulatory mechanism of Omi/HtrA2 in aged myocardial tissues remains elusive.

Further investigation of the mechanism of increased Omi/HtrA2 using a luciferase reporter assay indicated that the Omi/HtrA2 promoter core regions are situated within -1,205 to -838 bp and -146 to +93 bp. Furthermore, we confirmed that Heat shock factor 1 (HSF1) can bind to the Omi/HtrA2 promoter in the myocardium as a transcription factor [12]. These findings suggest that HSF1 is an important transcriptional factor that is involved in regulating Omi/HtrA2 mRNA levels. However, the role and expression of HSF1 and whether it regulates Omi/HtrA2 in the aging heart remain elusive.

In this study, we evaluated cardiac reserve and cardiac myocyte apoptosis in the aging mouse. We report that the overexpressing of the transcription factor HSF1 in cardiomyocytes up-regulates Omi/HtrA2 expression and contributes to mitochondrial apoptosis in aged cardiac myocytes. Our study investigated the regulatory mechanism of Omi/HtrA2 expression by HSF1 in the aging heart, and it provides a novel theory of HSF1 in myocardial apoptosis.

## RESULTS

### Cardiac reserve decreases in aged mice

The mouse aging model was constructed using four groups of mice based on ages: 4 months (young group), 12 months (middle age group), 18 months (presenium group), and 24 months (aging group) [13] and evaluated using different classic aging indexes: the mRNA and protein levels of aging-associated proteins p16, p53, and β-galactosidase The results showed that the mRNA levels of p16 and p53 increased with age (see Supplementary Figure 1A and 1B),. The protein levels of p16, p53, and β-galactosidase also increased with age (see Supplementary Figure 1C–1F). Mice 18 months and older showed poor in illustriousness and mobility, particularly those of age 24 months. Most of the mice with ages above 18 months had developed hearing loss. These findings supported that the mice used in this study were adequately grouped to simulate the process of physiological aging.

**Figure 1:**
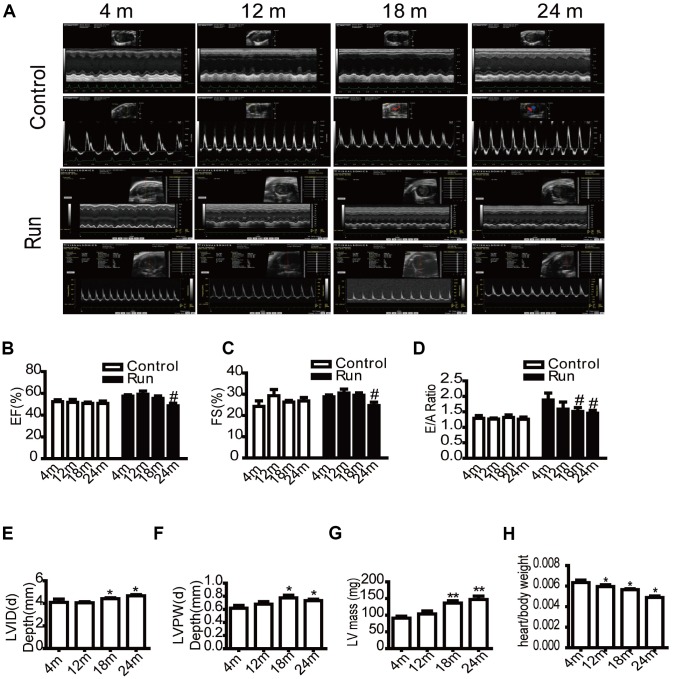
**The cardiac reserve function decreases in aging mice.** Echocardiography of the mice (**A**). Eject factor (EF, %) (**B**). Fractional shortening (FS, %) (**C**). The ratio of the early to late ventricular filling velocities (E/A ratio) (**D**). Left ventricular end-diastolic diameter (LVID(d), mm) (**E**). Left ventricular posterior wall end-diastolic thickness (LVPW(d), mm) (**F**). Left ventricular mass (LV mass, mg) (**G**). The ratio of the heart weight to total weight (**H**). Data are represented as mean +/- SEM. n = 6 per group. *P<0.05, **P<0.01 vs. 4 m. m=month.

To determine whether cardiac reserve had changed in aging mice, echocardiography was performed at different ages (4 months, 12 months, 18 months, and 24 months old). In our study, ejection fraction (EF) and fractional shortening (FS) reflected cardiac systolic function, and E/A ratio was tested for diastolic function. No significant differences were observed in EF, left ventricular FS, and E/A ratio at rest. However, after exercise load, the EF (Figure 1A and 1B), FS (Figure 1A and 1C) of the 24-month-old groups decreased compared to the 4-month-old group, and the E/A ratio (Figure 1A and 1D) of the 18- and 24-month-old groups decreased compared to the 4-month-old group, suggesting a decrease in the cardiac reserve of the aged group. Simultaneously, the results of echocardiography showed that the left ventricular end-diastolic diameter (LVID(d)) increased (Figure 1E), the left ventricular posterior wall end-diastolic thickness (LVPW(d)) increased (Figure 1F), and the mass of the left ventricle (LV mass) increased (Figure 1G) with aging. These findings were suggestive of left ventricular hypertrophy. We also measured the ratio of the heart weight to total weight to observe changes in the aging heart, which showed that the ratio decreased with age (Figure 1H). We measured the systolic blood pressure to evaluate changes in vascular function and found that the systolic blood pressure of the mice did not significantly change with age (Supplementary Figure 2). We also evaluated the levels of atherogenic process in aorta, there were no distinctly different pathological changes in aorta of aged rats by HE staining, Masson trichrome stain and sirus red stain (Supplementary Figure 3A–3C), however. To evaluate thoracic aorta endarterium morphology, thoracic aorta endothelium biomarkers CD31 was detected by immunohistochemical stain (IHC). It showed that the damage of intimal layer continuity, loss of some vascular endothelial cells, shallow staining, and enlarged junction space in aging (Supplementary Figure 3D). It dedicated that the endothelium injury in aged aorta.

### The rate of apoptosis of cardiomyocytes increases in aged mice via the mitochondrial apoptotic pathway and is negatively correlated to cardiac systolic function

Reduction of antioxidation ability and increasing oxidative stress occurred in aged heart. Levels of antioxidants showed that total antioxidant activity (T-AOC) decreased (Supplementary Figure 4A), SOD activity decreased with age (see Supplementary Figure 4B), and malondialdehyde (MDA) increased (Supplementary Figure 4C) in aged myocytes. The production of ROS in general and mitochondrial in particular was increased (Supplementary Figure 4D, 4E) in aging heart compared with 4 month old mice. The level of ATP in aging myocardium was decreased (Supplementary Figure 4F).

**Figure 2:**
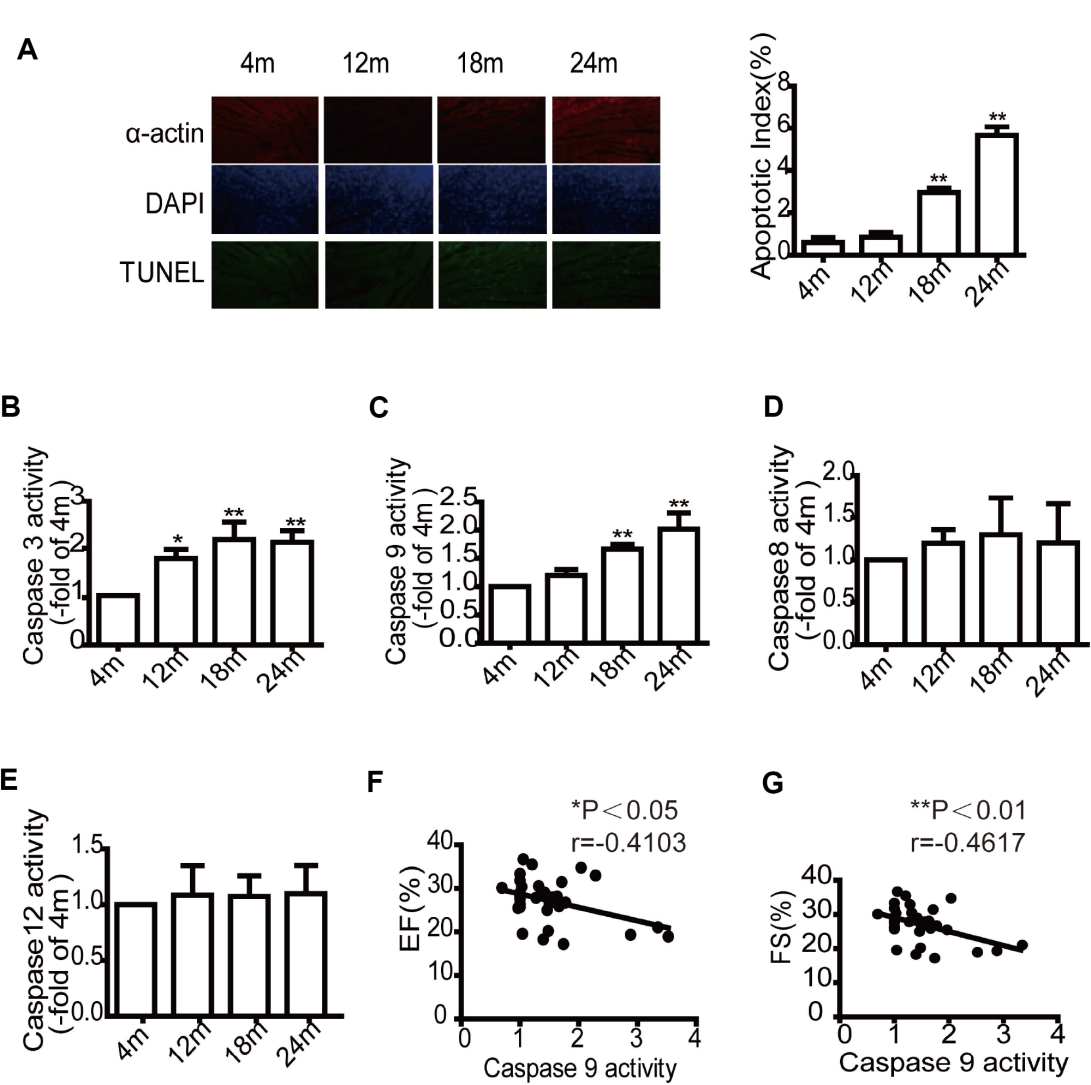
**Aging mice exhibit higher cardiomyocytes apoptotic rates and Caspase-9 activity but lower cardiac systolic function.** TUNEL labeling and apoptotic index of paraffin-embedded cardiac tissues (**A**). Evaluation of Caspase-3, Caspase-9, Caspase-8, Caspase-12 activity in mice of different ages (**B**–**E**), data are represented as mean +/- SEM. n = 6 per group. *P<0.05, **P<0.01 *vs.* 4m. m=month. The correlation analysis between the expression of Caspase- 9 activity and ejection fraction (EF, %) (**F**). The correlation analysis between the Caspase -9 activity and fractional shortening (FS, %) (**G**). n=32.

To evaluate the relevance of cardiomyocyte apoptosis and decreased cardiac reserve in aged mice, cardiomyocyte apoptosis in mice from different age groups was assessed by TUNEL staining myocardial tissue sections and determination of Caspase activity. The results demonstrated that the number TUNEL-positive cells increased with age (Figure 2A). In addition, Caspase-3 activity (a critical effector Caspase) in mouse cardiomyocytes also increased with age (Figure 2B). Caspase-9 activity (an initiator Caspase in the mitochondrial pathway) significantly increased in the older groups (18 and 24 months) (Figure 2C). However, Caspase-8 activity (an initiator Caspase of the extrinsic pathway) (Figure 2D) and Caspase-12 activity (an endoplasmic reticulum-specific stress-activated Caspase pathway) (Figure 2E) did not change. These results indicated that mitochondrial apoptosis is the main pathway in the aged heart. Caspase-9 activity was negatively correlated with left cardiac systolic function (EF, FS) after exercise (Figure 2F and 2G). However, there was also no correlation between Caspase-9 and diastolic function (Supplementary Figure 5).

### The upregulation of Omi/HtrA2 in aging myocardium is negatively correlated with the decline in left ventricular function and the ratio of heart weight to total weight

**Figure 3:**
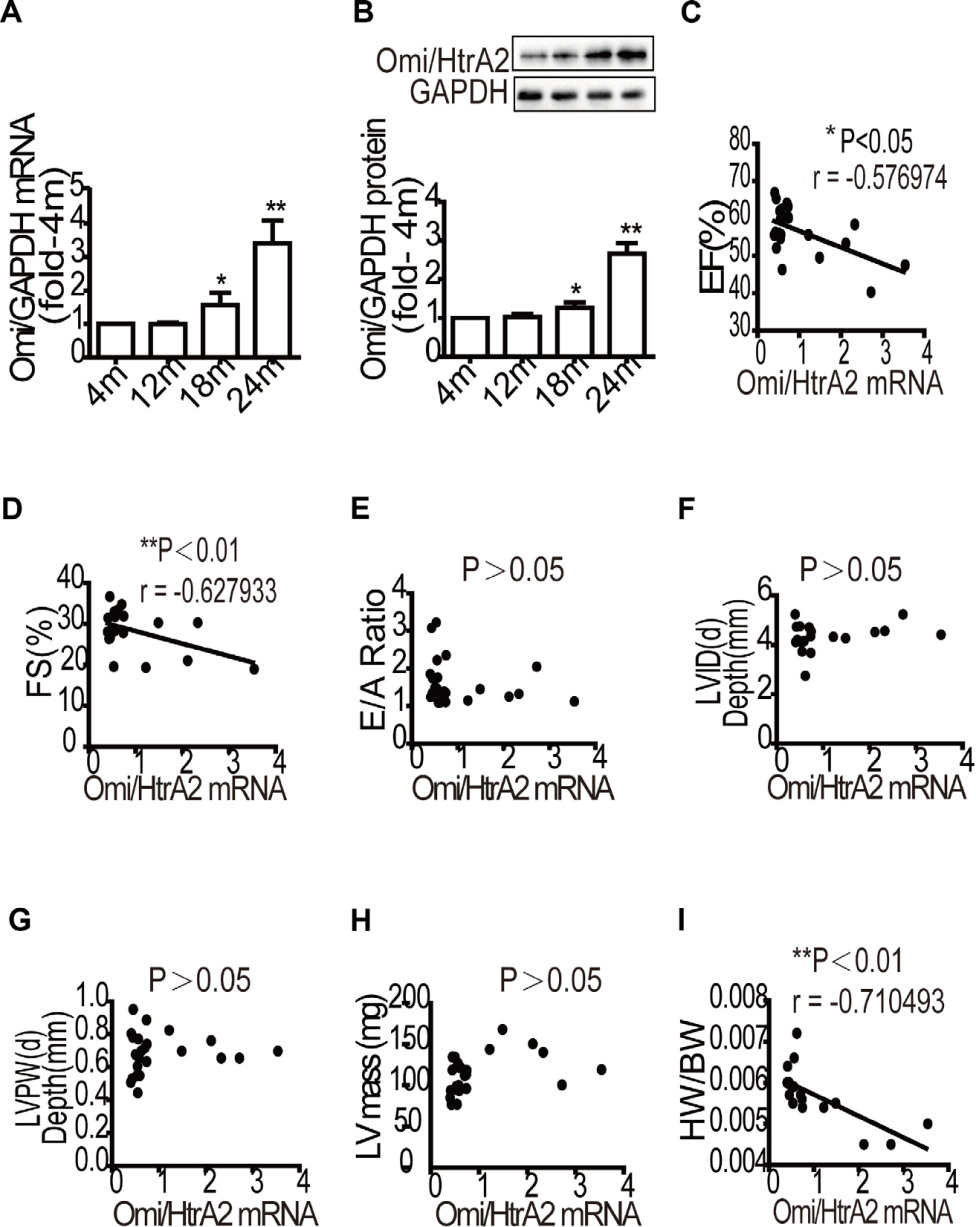
**Correlation analysis of Omi/HtrA2 mRNA expression and cardiac reserve function with aging**. The mRNA levels of Omi/HtrA2 were assessed by quantitative RT-PCR (**A**). Protein expression was evaluated by western blotting (**B**). Data are represented as mean +/- SEM, n = 6 per group. *P<0.05, **P<0.01 *vs.* 4m. m=month. A strong negative correlation between Omi/HtrA2 mRNA expression and ejection fraction (EF, %) was observed with aging (**C**). A significant linear negative correlation between Omi/HtrA2 mRNA expression and fractional shortening (FS, %) with aging (**D**). No significant linear correlation between Omi/HtrA2mRNA expression and E/A ratio (**E**). No significant linear correlation between Omi/HtrA2 mRNA levels and left ventricular end-systolic diameter (LVID, mm) was observed (**F**). The correlation analysis between Omi/HtrA2 mRNA levels and left ventricular posterior wall end-systolic thickness (LVPW, mm) (**G**). Correlation analysis between Omi/HtrA2 mRNA levels and left ventricular mass (LV mass, mg) (**H**). A significant linear negative correlation between Omi/HtrA2 mRNA expression and heart/body weight with aging (**I**).

To determine the expression of Omi/HtrA2 (a pro-apoptotic protein) in the aging heart, we detected its mRNA and protein levels by RT-qPCR and western blot analyses. The results showed that the mRNA and protein levels of Omi/HtrA2 significantly increased in the presenium group (18 months) and the older group (24 months) compared to the young group (Figure 3A and 3B). To evaluate the mRNA levels of Omi/HtrA2 and cardiac function in aging mice, we analyzed the correlation in the mRNA levels of Omi/HtrA2 as well as in the EF, FS, and diastolic index. The results showed that the mRNA levels of Omi/HtrA2 were negatively correlated with left ventricular systolic function (Figure 3C and 3D), but had no significant correlation with diastolic function (Figure 3E). We also analyzed Omi/HtrA2 mRNA, LVID(d), LVPW(d), and LV mass to explore the correlation between Omi/HtrA2 mRNA level and cardiac structure in aging mice, in which we found no significant correlation (Figure 3F–3H). However, the mRNA level of Omi/HtrA2 was negatively correlated with the ratio of heart weight to total weight (Figure 3I).

### Overexpression of HSF1 in myocardium promotes Omi/HtrA2 mRNA and protein expression by enhancing the promoter activity of Omi/HtrA2

**Figure 4:**
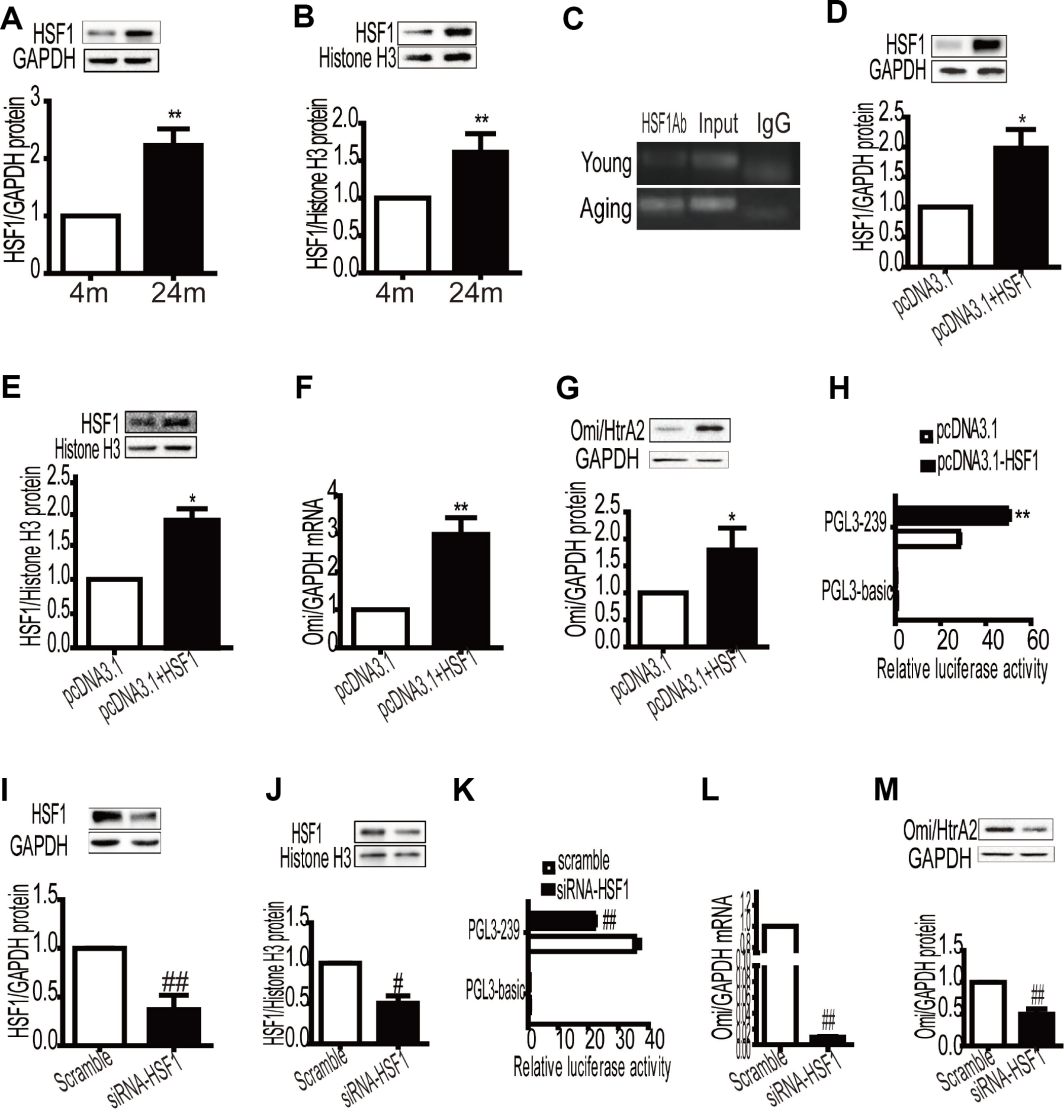
**Overexpression of HSF1 in the myocardium promotes the expression of Omi mRNA and protein levels by enhancing the activity of the promoter of Omi/HtrA2.** Total and nucleoprotein HSF1 expression was increased in aging myocardium, GAPDH, and histone H3 as internal control (**A**, **B**). Data are represented as mean +/- SEM. **P< 0.01 *vs*. young, n = 6 per group. The binding of HSF1 to the Omi/HtrA2 promoter increased in aging mice ©. Total and nucleoprotein HSF1 expression increased after transient transfection of pcDNA3.1-HSF1 (**D**, **E**). mRNA and protein levels of Omi/HtrA2 increased after transient transfection of pcDNA3.1-HSF1 (**F**, **G**). Relative luciferase activity of PGL-239 increased after transient transfection of pcDNA3.1-HSF1 (**H**). Values are means ± SEM, *P<0.05, **P<0.01 *vs.* pcDNA3.1, n = 3. Total and nucleoprotein HSF1 expression decreased after transient transfection of siRNA-HSF1 (**I**, **J**). Relative luciferase activity of PGL-239 decreased after transient transfection of siRNA -HSF1 (**K**). mRNA and protein levels of Omi/HtrA2 increased after transient transfection of pcDNA3.1-HSF1 (**L**, **M**), Values are means ± SEM, ^#^*P*<0.05, ^##^*P*<0.01 *vs.* scramble, n = 3.

HSF1 is a critical transcriptional factor correlated with chronic stresses and heart disease [14]. To investigate changes in HSF1 in the aging heart, HSF1 expression was detected by western blot analysis of heart tissues. High levels of the HSF1 protein were detected in the aged cardiac myocytes (Figure 4A). In our previous study, we found that HSF1 binds to the Omi/HtrA2 promoter as a transcription factor. To determine whether increased HSF1 is involved in Omi/HtrA2 expression, we detected the nucleoprotein in cardiomyocytes and also assessed changes in the binding ability of HSF1 to the promoter by ChIP. An increase in HSF1 levels in the nucleus was observed (Figure 4B), as well as binding to the Omi/HtrA2 promoter were observed in the aged heart (Figure 4C). To gain a deeper understanding of the mechanism involved in HSF1-based regulation of Omi/HtrA2, HSF1-overexpressing NIH3T3 cells were prepared by transiently transfecting pcDNA3.1-HSF1 (Figure 4D). HSF1 protein in the nucleus was regulated in the transfected NIH3T3 cells (Figure 4E). Overexpression of HSF1 increased the mRNA and protein levels of Omi/HtrA2 (Figure 4F and 4G), and the activity of the Omi/HtrA2 promoter was enhanced (Figure 4H).

To further confirm that HSF1 regulates Omi/HtrA2 expression through transcription activation, we detected the activity of the Omi/HtrA2 promoter after RNA interference of HSF1. The introduction of small interfering RNA (siRNA) resulted in the inhibition of HSF1 expression in the NIH3T3 cells (Figure 4I). The HSF1 nucleoprotein was downregulated (Figure 4J). Knocking down HSF1 resulted in a reduction in the activity of the Omi/HtrA2 promoter (Figure 4K), as well as the mRNA (Figure 4L) and protein levels of Omi/HtrA2 (Figure 4M).

### Overexpression of HSF1 induces mitochondria apoptosis by regulating Omi/HtrA2 expression in H9C2 cells

**Figure 5:**
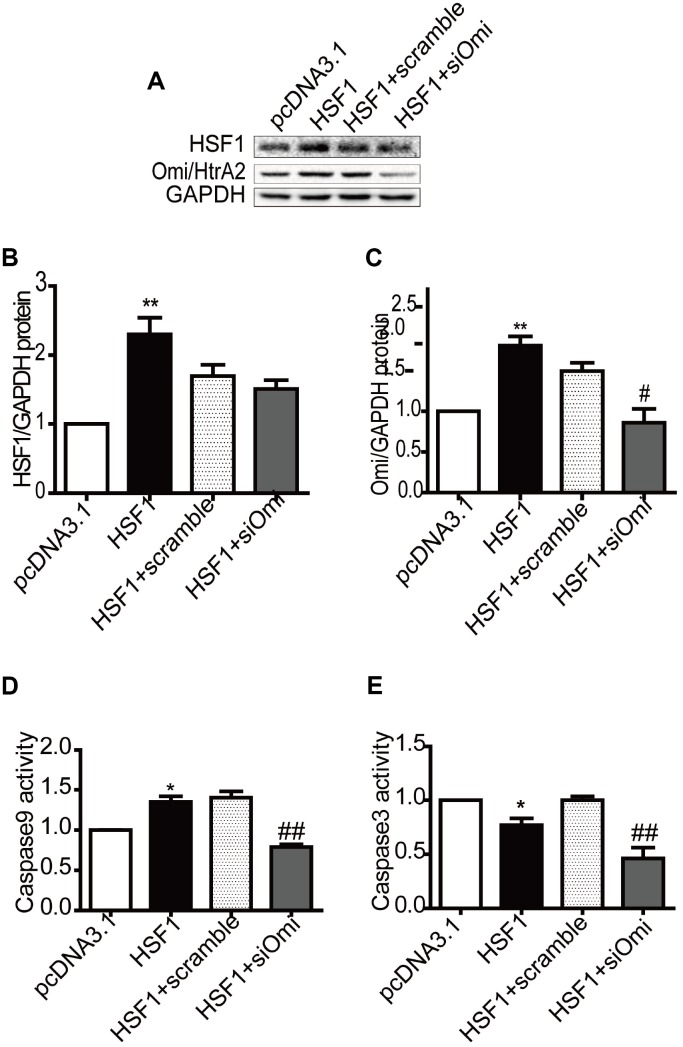
**Overexpression of HSF1 participates in mitochondrial apoptosis by regulating Omi/HtrA2 expression in H9C2 cells**. HSF1 expression increased after transfection of plasmid pcDNA3.1, which was not affected by RNAi Omi/HtrA2 (**A**, **B**). Omi/HtrA2 protein expression increased after transfection of plasmid pcDNA3.1, which decreased after RNAi Omi/HtrA2 (**A**, **C**). Caspase -9 activity was induced by HSF1 overexpression, which was reversed after transient transfection of si-Omi/HtrA2 (**D**). Caspase-3 activity was suppressed by HSF1 overexpression, but similar change was detected after RNAi using Omi/HtrA2 (**E**). Data are represented as mean +/- SEM. *P<0.05, **P<0.01 *vs.* pcDNA3.1, ^#^*P*<0.05, ^##^*P*<0.01 *vs.* HSF1+scramble, n = 4.

To determine the effects of HSF1 on Omi/HtrA2-mediated apoptosis in cardiomyocyte, we upregulated HSF1 by transiently transfecting the pcDNA3.1-HSF1 plasmid into cultured H9C2 cells, and HSF1 had no change after knockdown Omi/HtrA2 by RNA interference compared to that in the HSF1+scramble group (Figure 5A and 5B). Omi/HtrA2 expression increased in the pcDNA3.1-HSF1 group. Then, Omi/HtrA2 expression was silenced by RNA interference compared to that in the HSF1+scramble group (Figure 5A and 5C).

We demonstrated that Caspase-9 activity increased in the pcDNA3.1-HSF1 group, and the effect was suppressed by siRNA Omi/HtrA2 (Figure 5D). In addition, Caspase-3 activity was retained in the pcDNA3.1-HSF1 group, and similar change was observed after transfecting siRNA Omi/HtrA2 (Figure 5E).

## DISCUSSION

The present study describes the novel roles of HSF1 in regulating the pro-apoptosis protein Omi/HtrA2 in aged cardiomyocytes, which include the following: (i) using cardiac tissue, we demonstrated that HSF1 protein as well as its level in the nucleus were increased in the aged heart; (ii) overexpression of HSF1 induces

Omi/HtrA2 expression by enhancing the promoter activity of Omi/HtrA2 in aged cardiomyocytes; (iii) overexpression of HSF1 enhances mitochondria apoptosis by upregulating Omi/HtrA2 in vivo. Taken together, these results indicate that HSF1 as a transcriptional factor participates in myocardial mitochondria apoptosis by enhancing the promoter activity of the pro-apoptosis gene Omi/HtrA2 in aged cardiomyocytes.

Aging is a vital risk factor that increases the risk of developing cardiovascular decrease. A decrease in cardiac reserve [15], high sensitivity to ischemia reperfusion injury [16], and stress load [17], are characteristics of the elderly that reduce the quality of life. Cardiomyocyte apoptosis increases the risk of cardiovascular disease in the aged heart [13]. In this study, four groups of mice of different ages were used to simulate the process of physiological aging. We demonstrated that cardiac functional reserve decreased in the aged group after exercise, whereas no significant change was observed at the resting state. Aorta endothelium damaged in aged mice. Oxidant/antioxidant imbalance was more pronounced, In particular, mitochondrial oxidative stress increased. Apoptosis increased mainly via the mitochondrial pathway and was correlated to cardiac functional reserve, particularly the left heart diastolic function. These findings indicate that mitochondrial pathway apoptosis contributes to deficient cardiac functional reserve.

Several studies have indicated that the mitochondria-dependent apoptosis signaling pathway is more active in multiple organs of the elderly, for instance, muscle [18], heart [19]. Our previous study showed that the increased expression of Omi (also known as HtrA2) enhances MI/R injury in aged hearts [9] by inducing myocardial mitochondrial apoptosis [11].

Omi/HtrA2 is a serine protease located in the eukaryotic mitochondria, belonging to the high temperature requirement (HtrA) family. Studies have shown that mice with the motor neuron degeneration 2 (mnd2) mutation [20] and mice with Omi/HtrA2 knockout [21] exhibit a premature phenotype, which include osteoporosis, muscle atrophy, cardiac enlargement, and shortened life expectancy. Kang et al. reported that Omi/HtrA2 can regulate the protein quality of the mitochondrial intermembrane space [22], which is important for maintaining mitochondrial homeostasis. Disruption of mitochondrial homeostasis may be the reason for neurodegenerative disorders and premature senescence in non-nerve tissues [23].

In this study, we found that Omi/HtrA2 mRNA and protein levels increased in the presenium, with an even greater increase in the elderly group compared to the young group. The results were consistent with that of Caspase-9 activity. We also observed that the mRNA level of Omi/HtrA2 was negatively correlated with left ventricular systolic function after exercise and the ratio of heart weight to total weight. This study supported our previous findings that Omi/HtrA2 mRNA and protein levels increased with age and suggest that alterations in Omi/HtrA2 transcription and protein levels are closely associated with cardiac reserve by inducing mitochondrial pathway apoptosis. However, the regulatory mechanism of Omi/HtrA2 transcription in the aged heart remains elusive.

Many factors impact on the expression of Omi/HtrA2. Prior study showed that hypoxia-inducible factor (HIF)-1alpha inhibits Omi/HtrA2 expression and suppresses hepatocellular carcinoma cell apoptosis [7]. We previously confirmed that HSF1 can bind to the promoter region of Omi/HtrA2 in the myocardium by ChIP assay. This study confirmed that HSF1 as a transcription factor can regulate Omi/HtrA2 expression by showing that the nucleoprotein expression of HSF1 in the aging myocardium increased, along with the binding to the Omi/HtrA2 promoter. We also confirmed that overexpressed HSF1 can increase Omi/HtrA2 mRNA and protein levels by enhancing the activity of the Omi/HtrA2 promoter. These findings suggest that the activation of Omi/HtrA2 by HSF1 may be one of the mechanisms of Omi/HtrA2 overexpression in the aged myocardium.

HSF1 is a major factor in regulating the expression of heat shock proteins, plays an important role in maintaining the accurate folding of proteins [14] and cellular homeostasis [24], and participates in cellular processes such as the stress response, aging, and carcinogenesis [14]. HSF1 performs very important functions in cardiovascular disease. HSF1 protects cardiomyocyte from I/R injury [25] and HSF1 phosphorylation, which is activated by angiotensin II (ANG II), resulting in cardiac hypertrophy [26]. Furthermore, ANG II promotes insulin-like growth factor II receptor (IGF-IIR) expression and cardiomyocyte apoptosis by inhibiting HSF1 via c-Jun N-terminal kinase (JNK) activation and sirtuin 1 (SIRT1) degradation [27]. HSF1 expression increases in muscle tissue during aging [28]; however, HSF1-controlled chaperone capacity is suppressed in aged muscle cells [29]. This suggests that HSF1 has a negative effect during aging. We have shown that HSF1 as a transcription factor of Omi/HtrA2 enhances pro-apoptotic Omi/HtrA2 expression, thereby inducing cardiomyocyte apoptosis. HSF1 inhibits cardiomyocyte apoptosis but enhances mitochondrial apoptosis by upregulating Omi/HtrA2 expression.

In summary, HSF1 enhances cardiomyocyte mitochondria -mediated apoptosis by activating Omi/HtrA2 transcription, and Omi/HtrA2 expression influences the cardiac function reserve of the aging heart.

## MATERIALS AND METHODS

### Animals

The C57BL/6 male mice at various ages, namely, 4 months (young group), 12 months (middle age group), 18 months (presenium group), and 24 months (aging group) were purchased from Suzhou Ai Er Mai Te Technology Co., Ltd. (Suzhou, China). All animal experiments were conducted according to the Guide for the Use and Care of Animals of the National Institutes of Health (NIH Publication No. 85-23, revised 1996). All animals had free access to standard laboratory rat chow and tap water. Until parturition, rats were housed individually in a room at constant temperature (24 °C) and under a 12-h light–dark cycle.

### Cell lines, cell culture, and transfection

The NIH3T3 cell line was purchased from the Basic Medical Cell Center of Peking Union Medical College (Beijing, China). The NIH3T3 cells were grown in Dulbecco’s modified Eagle’s medium (high glucose) with 10% calf serum. The cells were cultured at 37°C and 5% CO_2_. Transfection was performed using Lipofectamine 3000 reagent (Invitrogen, USA) according to the manufacturer’s instructions. Cells were inoculated in six-well plates and divided into two groups: pcDNA-3.1 (no insert) and pcDNA3.1-HSF1. The medium was aspirated and replaced with serum-free medium without antibiotics 4h before transfection. Lipofectamine 3000 (3.75 μL) was diluted with 125 μL Opti-MEM and 2.5 μg plasmid, 5 μL p3000 were diluted with 125 μL Opti-MEM® reduced-serum medium (Gibco, Carlsbad, CA, USA). The mixtures were incubated for 20 min at room temperature and then added to the cells with antibiotic-free complete medium. Cell lysates were harvested 72h after transfection.

### siRNA oligos and transfection

Transfection of NIH3T3 cells was performed using Lipofectamine RNAiMAX (Thermo Scientific, USA) according to the manufacturer’s instructions. Cells were inoculated in six-well plates and divided into two groups: scramble/HSF1+scramble; si-Omi/HSF1+si-Omi. HSF1-siRNA5'-GGACACAACCGGAGCCCAA-3' (25pmol), and RNAiMAX were diluted with Opti-MEM (#31985; Gibco, Carlsbad, CA, USA). The mixtures were incubated for 20 min and then added to the cells. The medium was aspirated and replaced with Dulbecco’s modified Eagle’s medium (high glucose) supplemented with 10% calf serum. The cells were harvested 72 h after transfection.

### Western blot analysis

Myocardium tissues were harvested for Western Blot following standard protocol. Nucleoprotein was extracted according to the Nuclear and Cytoplasmic Protein Extraction Kit (P0027, Beyotime Institute of Biotechnology, Suzhou, China). 50 μg/lane protein or 20 μg/lane nucleoprotein Proteins were run on 10% SDS-PAGE, and then electrotransferred onto polyvinylidene difluoride membranes. The membranes were incubated with the primary antibodies anti-HSF1(#4356; 1:1000; Cell Signaling Technology, Danvers, MA, USA), anti-Omi/HtrA2(#2176; 1:1000; Cell Signaling Technology, Danvers, MA, USA), anti-GAPDH(#2118; 1:1000; Cell Signaling Technology, Danvers, MA, USA), or anti-Histone H3 (#4499; 1:1000; Cell Signaling Technology, Danvers, MA, USA) overnight at 4°C. After incubation with the corresponding secondary antibodies, the membrane was developed with a chemiluminescent substrate (Bio-Rad), and protein bands were measured using the ImageJ software.

### Quantitative PCR

The total RNA of was isolated using TRIzol™ reagent (Invitrogen; USA) according to the manufacturer’s instructions. Total RNA was reverse-transcribed using a PrimeScript™ RT Master Mix (#RR036A; Takara), gene expression was assessed by real-time PCR using a SYBR PrimeScript™ RT-PCR kit (#RR820A; Takara) as previously described [12]. Primers for QRT-PCR (SYBR) were designed and synthesized as follows: Omi-F: 5′-ATCTCCTTTGCCATCCCTTC-3′; Omi-R: 5′-GGTCAGCATCATCACTCCAA-3′; p16-F: 5′-CGT GTCTAGCATGTGGCTTT-3′; P16-R: 5′-GCCTTCGC TCAGTTTCTCAT-3′; p53-F: 5′-CGTAAACGCTTCG AGATGTTC-3′; P53-R: 5′-GCCCTTCTTGGTCTTCA GGT-3′; HSF1-F: 5′-GGCAGTACCTTGGATCAGGA-3′; HSF1-R: 5′-CAAGTGTGGCTGTGAAGCTG -3′;.

### Luciferase activity assay

Luciferase activity assay was performed as previously described [12]. The NIH3T3 cells were inoculated in 96-well plates and then transfected as described in the above methods section using Lipofectamine 3000 reagent (Invitrogen, USA). The cell lysates were harvested 48h after transfection. The Dual-Glo® Luciferase Assay System (#E2920; Promega, Madison, WI, USA) was used to test the firefly luciferase and Renilla luciferase activity using a GloMax® 96-well microplate luminometer (Promega, Madison, WI, USA) for measurements. Relative luciferase activity (RLA) was the ratio of firefly luciferase to Renilla luciferase.

### Echocardiographic and hemodynamic measurements

Left ventricular end-diastolic diameter (LVID(d)), Left ventricular posterior wall end-diastolic thickness (LVPW(d)), and Left atrioventricular mass (LV mass) were measured and recorded on the left heart long axis section (Vevo 2100, Visual Sonic, Canada). Eject factor (EF) and Fractional shortening (FS) were obtained by M-type ultrasound, and all data were measured three times and averaged. E-peak and late-filling A were evaluated using a mitral valve flow chart. The E/A ratio was calculated to reflect left ventricular diastolic function. LVID(d), LVPW(d) and LV mass were measured without any treatment or exercise load (using animal experiment treadmill at a runway speed of 15 m/min and exercise time of 10 min), and EF, FS, and E/A ratio were recorded.

### Detection of mouse blood pressure

We used the mouse tail arterial pulse pressure method to measure blood pressure; the sensor was set at the tail of the mouse, and blood pressure was determined by monitoring the blood flow signal while inflating and deflating the tail artery and releasing the pressure.

### Terminal-deoxynucleotidyl transferase mediated nick end labeling (TUNEL) staining of mouse hearts and determination of Caspase protease activity

The myocardial tissues were perfused with 4% paraformaldehyde in PBS, and then embedded in paraffin. TUNEL staining (In Situ Cell Death Detection Kit, Roche) was performed according to the manufacturer's instructions. The percentage of apoptotic nuclei in each slide was used to determine the apoptotic index. The concentration of the proteins extracted from myocardial tissue was determined using the Bradford assay. Caspase protease activity was determined with a colorimetric assay kit according to the manufacturer’s instructions. Briefly, a mixture consisting of 50 μL of the 2×assay buffer, 30 μL of the sample supernatant, and 10 μL of ddH2O was prepared. Then, the Ac-DEVD-AFC/Ac-IETD-AFC/AC-IEHD-AFC/AC-ATAD-AFC substrate (10μL) was added to each well of a 96-well microplate. The absorbance of each well was then measured on a fluorescent microplate reader (Excitation: 400 nm and Emission: 508 nm).

### Chromatin immunoprecipitation (ChIP) assay

ChIP assay (Magna ChIP G Tissue Kit #17-20000; Millipore EZ-ChIP, Darmstadt, Germany) was conducted as previously reported [12], according to the manufacturer's instructions. Mouse myocardial tissues was crosslinked with 1% formaldehyde, then sonicated to shear chromatin DNA to sizes within the range of 200-500 bp. The DNA fragments were immunoprecipitated with rabbit α-mouse IgG as control and also with antibodies specific to HSF1 (#4356; Cell Signaling Technology, Danvers, MA, USA). Immunoprecipitation was performed overnight at 4°C. The protein/DNA complexes and reverse crosslinks of protein/DNA complexes to free DNA were eluted, and the immunoprecipitated chromatin was amplified using primers corresponding to the Omi/HtrA2 promoter. Primers were as follows: Omi promoter-F: 5′-GCTACCGTCGTGCCCTGCTT-3′; Omi promoter-R: 5′-ATGCCCGAAGGCTCCAGTTT-3′

### Immunohistochemical stain (IHC)

After being embedded in paraffin, all samples of thoracic aorta tissue cubes were sectioned into three 4-mm-thick slices and stained with hematoxylin and eosin (HE), Masson’s trichrome and Sirius red stain.

Thoracic aorta tissue samples (1cm^3^) were fixed with para-formaldehyde and embedded in paraffin. 4 μm sections were incubated in a dilution of rabbit anti- CD31 antibody (ab182981,1:2000, Abcam, USA) overnight at 4°C. Immuno-reactivity was detected by goat anti-rabbit IgG (zsgb, CHN). After dehydration, all sections were observed and photographed using a Nikon microscope fitted with a camera (NIKON Corporation, JPN).

### Antioxidant activity assay and Free radicals

Total antioxidant capacity (T-AOC) (ABTS method), SOD activity (WST-1 method) and malondialdehyde (MDA) (TBA method were detected according to the manufacturer’s instructions by corresponding kits (A015-2-1, A001-3, A003-1-2, Nanjing Jiancheng Bioengineering Institute, Nanjing, China). The absorbance was measured using a microplate reader at corresponding wavelength. The T-AOC, SOD activity and MDA were calculated with provided formulas separately.

### Assessment of reactive oxygen species (ROS)

The levels of ROS in general was determined by reactive oxygen species Assay Kit (E004-1, Nanjing Jiancheng Bioengineering Institute). The reaction solution was mixed with samples according to the manufacturer’s instructions. The cells were washed with D-Hank’s and incubated with 2′,7′dichlorofluorescein diacetate (DCFH-DA) at 37°C for 20 min. Then DCF fluorescence distribution of cells was detected by fluorospectrophotometer analysis at an excitation wavelength of 488 nm and at an emission wavelength of 535 nm.

The levels of ROS in mitochondrial was evaluated with the same methods after Cells were collected and treated according to the manufacturer's instructions (Tissue Mitochondria Isolation Kit, Beyotime).

### ATP test

ATP Assay Kit (S0026, Beyotime) was measured according to the manufacturer’s instructions. The reaction solution was mixed with samples at room temperature for 5 minutes, and the relative light unit (RLU) was measured using a luminometer.

### Statistical analysis

All values in the text and figures are expressed as the mean ± SEM. All data (except for western blot density) were subjected to ANOVA, followed by post hoc Bonferroni correction. Western blot densities and RT-qPCR were analyzed using the Kruskal-Wallis test followed by Dunn’s post hoc test. Probabilities of 0.05 or less were considered statistically significant.

## Supplementary Material

Supplementary Figures
