# Supplementary material for: Heat shock factor 1-mediated transcription activation of Omi/HtrA2 induces myocardial mitochondrial apoptosis in the aging heart
Source: Aging (Albany NY). 2019 Oct 18;11(20):8982–97. doi: 10.18632/aging.102361 (PMC6834417; doi:10.18632/aging.102361)
Supplement: Supplementary Figures [file aging-11-102361-s001.pdf]

SUPPLEMENTARY FIGURES

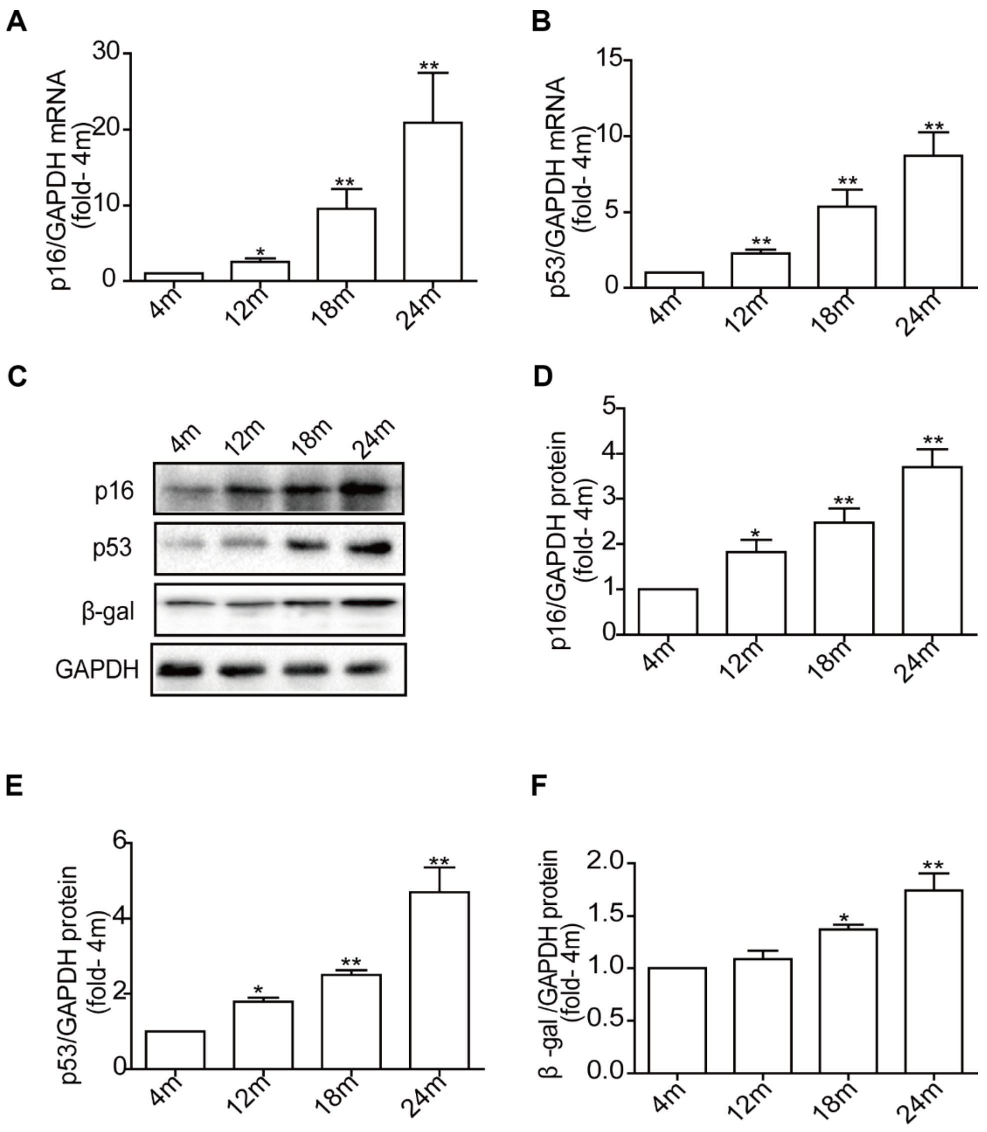

Supplementary Figure 1. Identification of the aging mice. mRNA expression of p16 (**A**) and p53 (**B**) were increased, protein expression of p16, p53 and β-gal (**C**–**F**) were increased with aging. Data are represented as mean +/- SEM. n = 6 per group. * $p < 0.05$, ** $p < 0.01$ *vs.* 4m. m=month.

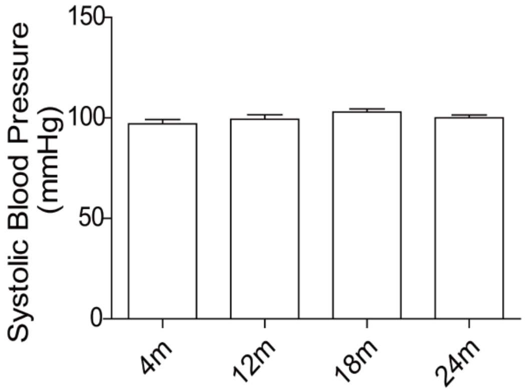

Supplementary Figure 2. Systolic blood pressure of the mice. Compared with 4m group, Systolic blood pressure had no significant changed with aging. Data are represented as mean +/- SEM. n = 6 per group. m=month.

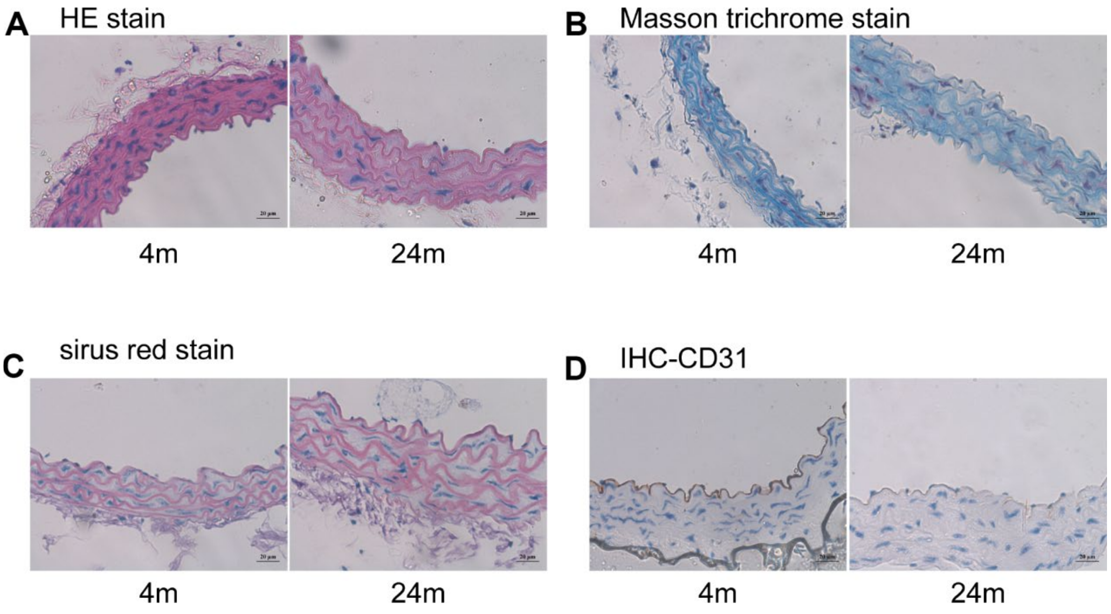

Supplementary Figure 3. Thoracic aorta endothelium damaged in aged mice. (**A**) HE staining of thoracic aorta; (**B**) Masson trichrome stain of thoracic aorta; (**C**) Sirius red stain of thoracic aorta; (**D**) immunohistochemistry staining of endothelium marker CD31 of thoracic aorta. m=month. n = 6 per group. bar=20μm.

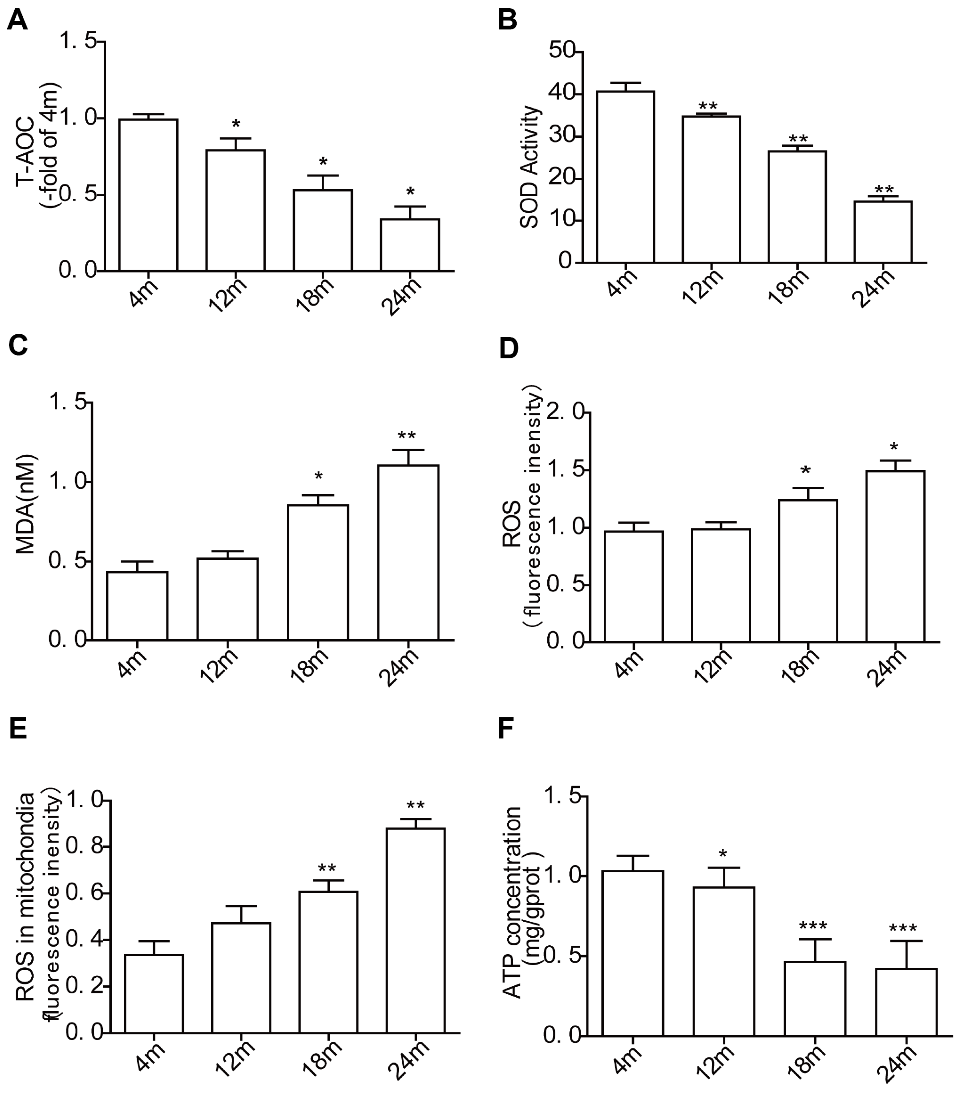

Supplementary Figure 4. Antioxidation ability decreased and oxidative stress increased in aged heart. Total antioxidant activity (T-AOC) decreased (**A**), SOD activity decreased with age (**B**), and malondialdehyde (MDA) increased (**C**) in aged myocytes. The production of ROS in general (**D**) and mitochondrial in particular (**E**) was increased in aging heart, ATP was decreased in aging myocardium (**F**), compared with 4 month old mice. Data are represented as mean +/- SEM. n = 6 per group. * $p < 0.05$, ** $p < 0.01$, *** $p < 0.001$ *vs.* 4m. m=month.

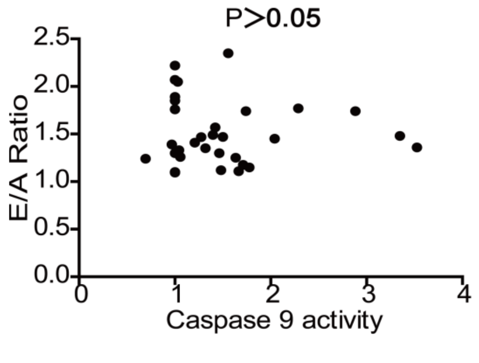

Supplementary Figure 5. No correlation between caspase-9 activity and diastolic function. n=32.
